# Physiological and Morphological Responses of the Temperate Seagrass *Zostera muelleri* to Multiple Stressors: Investigating the Interactive Effects of Light and Temperature

**DOI:** 10.1371/journal.pone.0076377

**Published:** 2013-10-04

**Authors:** Paul H. York, Renee K. Gruber, Ross Hill, Peter J. Ralph, David J. Booth, Peter I. Macreadie

**Affiliations:** 1 School of the Environment, University of Technology, Sydney, Broadway, NSW, Australia; 2 Centre for Tropical Water and Environmental Research Centre, James Cook University, Cairns, QLD, Australia; 3 Office of Environment and Heritage, Sydney, NSW, Australia; 4 Oceans Institute, University of Western Australia, Crawley, WA, Australia; 5 Plant Functional Biology and Climate Change Cluster, University of Technology, Sydney, Broadway, NSW, Australia; 6 Centre for Marine Bio-Innovation, School of Biological, Earth and Environmental Sciences and Sydney Institute of Marine Science, the University of New South Wales, Sydney, NSW, Australia; University of Sydney, Australia

## Abstract

Understanding how multiple environmental stressors interact to affect seagrass health (measured as morphological and physiological responses) is important for responding to global declines in seagrass populations. We investigated the interactive effects of temperature stress (24, 27, 30 and 32°C) and shading stress (75, 50, 25 and 0% shade treatments) on the seagrass *Zostera muelleri* over a 3-month period in laboratory mesocosms. *Z. muelleri* is widely distributed throughout the temperate and tropical waters of south and east coasts of Australia, and is regarded as a regionally significant species. Optimal growth was observed at 27°C, whereas rapid loss of living shoots and leaf mass occurred at 32°C. We found no difference in the concentration of photosynthetic pigments among temperature treatments by the end of the experiment; however, up-regulation of photoprotective pigments was observed at 30°C. Greater levels of shade resulting in high photochemical efficiencies, while elevated irradiance suppressed effective quantum yield (ΔF/F_M_’). Chlorophyll fluorescence fast induction curves (FIC) revealed that the J step amplitude was significantly higher in the 0% shade treatment after 8 weeks, indicating a closure of PSII reaction centres, which likely contributed to the decline in ΔF/F_M_’ and photoinhibition under higher irradiance. Effective quantum yield of PSII (ΔF/F_M_’) declined steadily in 32°C treatments, indicating thermal damage. Higher temperatures (30°C) resulted in reduced above-ground biomass ratio and smaller leaves, while reduced light led to a reduction in leaf and shoot density, above-ground biomass ratio, shoot biomass and an increase in leaf senescence. Surprisingly, light and temperature had few interactive effects on seagrass health, even though these two stressors had strong effects on seagrass health when tested in isolation. In summary, these results demonstrate that populations of *Z. muelleri* in south-eastern Australia are sensitive to small chronic temperature increases and light decreases that are predicted under future climate change scenarios.

## Introduction

Seagrasses provide many essential ecosystem services: they stabilise shorelines and prevent coastal erosion [1]; they play a key role in nutrient cycling worth US$19,002 ha^-1^ yr^-1^ [2]; they provide critical habitat for thousands of fish, bird, and invertebrate species [3]; they support ~50% of the world’s fisheries through provision of nursery habitat [4]; and they are now considered to be the most powerful carbon sinks on the planet [5]. However, seagrasses are currently facing a global crisis [6]; 29% of the world’s seagrasses have disappeared [7,8], and 14% of all seagrass species are at risk of extinction [7].

Seagrass loss can be attributed to many forms of disturbance, both natural and anthropogenic. The majority of declines are attributed – directly (e.g. dredging) or indirectly (e.g. eutrophication) – to low light stress [9]. Seagrasses have unusually high light requirements; 10-37% of surface irradiance vs. 0.1-1% for most other marine acrophytes [10], which makes them highly vulnerable to deterioration in water clarity. Understanding light thresholds for seagrass survival is therefore critical for effective management of seagrass habitats.

Over the past few decades there has been progress towards understanding responses of seagrasses to light limitation at the plant biochemistry level [11,12] and how these changes influence the distribution of seagrass at the landscape scale [13,14,15]. However, there is still little information on how light limitation interacts with other stressors – particularly increases in water temperatures – to influence seagrass health. Such information is critical for developing better predictions of future seagrass distributions, especially under climate change scenarios [16].

Photosynthetic processes of seagrasses are highly responsive to light limitation and thermal stress, with sharp reductions in photosynthetic efficiencies occurring when thresholds for light starvation and thermal tolerance are exceeded [17,18,19]. Metabolic imbalances, whereby the photosynthesis to respiration ratio (i.e. P:R) is less than 1, typically cause declines in seagrass growth and re-mobilization of plant carbon storage reserves during periods of light starvation [11,20,21,22]. This can cause seagrasses to shift from being carbon ‘sinks’ to carbon ‘sources’, which can serious implications for the ability of seagrass ecosystems to offset carbon emissions.

This study is one of a small but growing number that investigate the interaction of multiple stressors on seagrass. The goal of this study was to investigate the interactive effects of light limitation and elevated temperature stress on seagrass health; measured as morphological and physiological parameters of seagrass that are known to respond to these stressors such as photosynthetic efficency, photoprotective capability, leaf morphology, and partitioning of above and below ground biomass [23]. We focus on *Zostera muelleri* (syn *Zostera capricorni*) Irmisch ex Asch, which is the dominant seagrass species (in terms of total area) [24] in temperate and sub-tropical south-eastern Australia. Regional climate models for this region predict increases in water temperatures and reduced light availability in coastal areas [25]. Regression modelling indicates that these stressors have already reduced seagrass biomass in the region during the past 16 years of monitoring [26]. Based on recent studies involving human-induced stressors [27], we predicted that light limitation and thermal stress will have a negative, synergistic effect on seagrass health.

## Materials and Methods

### Seagrass specimen collection and pre-treatment

**Figure 1:**
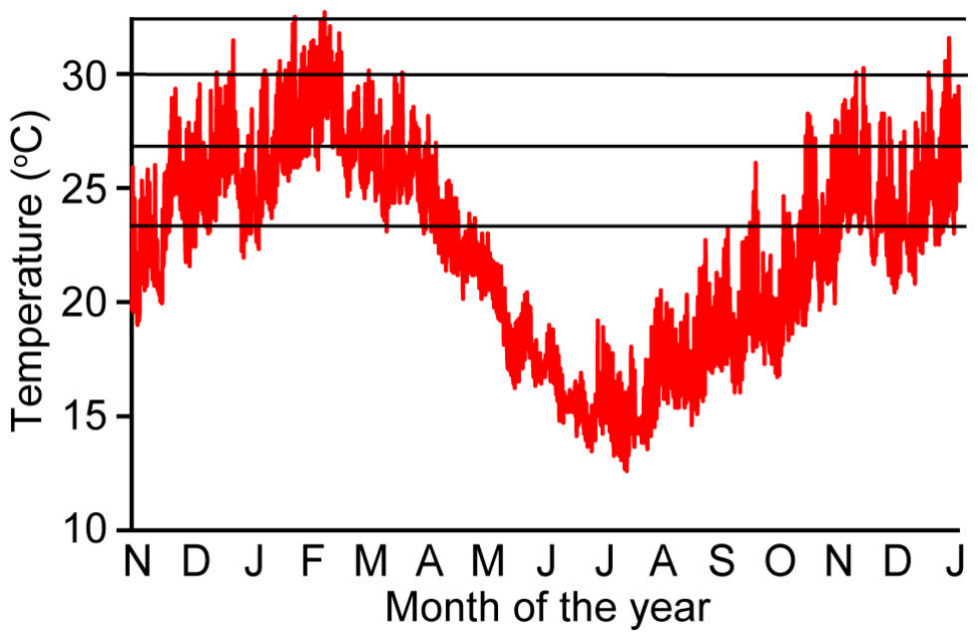
Temperature profiles in Lake Macquarie. Seasonal and diurnal temperature variation measured by Hobo loggers over 15 months (November 2010 – January 2012) at Sunshine, Lake Macquarie. Horizontal bars represent the four temperature treatments used in the experiment.

*Zostera muelleri* was collected from shallow sub-tidal seagrass beds at Cams Wharf (33°07’33” S 151° 36” 49” E) in Lake Macquarie, a large, wave-dominated barrier estuary in New South Wales (NSW), Australia [28]. Water temperature in these shallow seagrass beds within Lake Macquarie varies greatly both diurnally and seasonally, ranging from 23-33°C (mean of 26.5°C) in summer to 12-19°C in winter (Figure 1).

Seagrass was collected in large intact sods (^≈^ 300 x 300 mm) containing leaves, rhizomes and sediment and transported in aerated seawater to the laboratory. The seagrass was then sliced into 150 mm x 100 mm segments and much of the sediment was removed. Seagrass segments were then replanted in a media of 70% sand: 30% loam in 500 mL plastic punnets (175mm x 120 mm x 45 mm) and a light coating of washed beach sand was sprinkled on top to stabilise the sediments. Effort was made to maintain seagrass belowground biomass intact during transplanting, which meant that the number of shoots varied among tanks (range: 17-76 shoots, mean: 43); however, tanks were randomised among treatments so that this variation did not bias the results. Furthermore, to standardise the results, rates of mortality, leaf loss, and declines in biomass are represented as a proportion of the original amounts present within each tank.

Prior to the start of the experiment, the seagrasses were allowed to acclimate over a week-long period. Experimental units (seagrasses in punnets) were kept in holding tanks in a glasshouse (temperature range ^≈^ 18-28°C) with ambient light levels and monitored daily for signs of stress (e.g. leaf loss, changes in Effective Quantum Yield of PSII - see below). They were then relocated to the experimental temperature-controlled room (average of 18 ± 2°C) and placed into clear plastic 10 L tanks (260 mm x 160 mm x 180 mm) filled with natural seawater (24°C). In order to avoid thermal shock, temperatures were increased slowly over 3 days until they reached their temperature treatment levels.

### Experimental Design

Decreases in available light have long been recognized as one of the main contributors to seagrass loss in Australia [29], and poor water clarity is likely to continue (and worsen) in coming decades. Light levels in this study were chosen to correspond to three levels (high, mid, low) that should be tolerable to *Z. muelleri*, plus one treatment below the compensation point (based on a growing season light level of 1000 *µ*E m^-2^ s^-1^ and 10% of surface light requirement). Light was applied with 400 W metal-halide lamps fitted with light-diffusing covers. Between the light source and the water surface, clear, 0.15, 0.3, and 0.6 graduated neutral density filters (LEE Filters) were installed to create four levels of shading: 0% (231 ± 18 µmol photons m^-2^ s^-1^), 25% (162 ± 9 µmol photons m^-2^ s^-1^), 50% (112 ± 6 µmol photons m^-2^ s^-1^), and 75% (47 ± 2 µmol photons m^-2^ s^-1^), respectively. Lights were set on a 12 hour cycle (see Figure S1 for a schematic of the experimental setup).

In the shallow coastal waters colonized by *Z. muelleri*, mean temperatures can vary considerably with water depth, river inflow, and rates of ocean flushing. Temperature treatments were selected to represent low and mean growing season levels in Lake Macquarie NSW (see Figure 1) (24 and 27°C, respectively), and predicted future temperatures under a warming scenario for 2050 [25] (30 and 32°C). Three replicates of each treatment were haphazardly assigned to each shade treatment to produce a fully-orthogonal experimental design. Temperature in each tank was controlled using a 25 W submersible aquarium heater (23.6 ± 0.1 °C, 26.7 ± 0.1 °C, 29.2 ± 0.1 °C, 31.7 ± 0.2 °C for respective treatments). Salinity and temperature were monitored twice daily (WTW 315i Conductivity meter) and adjusted where necessary. When salinities exceeded 35, RO water was added to maintain a natural range (30-35). All tanks were partially drained (60-75%) and re-filled (6 L per tank) three times per week.

### Chlorophyll fluorescence

Pulse amplitude modulated (PAM) fluorometry was used to measure the diel effects of light and temperature manipulation on seagrass maximum (F_V_/F_M_) and effective quantum yield (ΔF/F_M_′) of photosystem II (PSII) (Mini-PAM; Walz GmbH, Germany). Measurements were taken three times weekly (first 2 months of the experiment) and twice weekly (third month). F_V_/F_M_ was measured before the lights turned on at 0800 h, while ΔF/F_M_′ was measured during the light period at 1500 h. Quantum yield readings were taken after the application of a saturating pulse of light (measuring intensity of <0.15 µmol photons m^-2^ s^-1^, saturation intensity of >4000 µmol photons m^-2^ s^-1^, saturation width of 0.8 s, and an output gain of 10) on the most central mature leaf of seagrass shoots.

In order to provide detailed information on the photochemical condition of PSII, including the redox state of the primary (Q_A_) and secondary (Q_B_) electron acceptors and the plastoquinone (PQ) pool [30,31], fast induction curves (FICs) were generated using a Plant Efficiency Analyser (PEA) (Hansatech Instruments, King’s Lynn, UK). On Days 1, 27, 53 and 92 of the experiment, 3-8 cm of the central blade of seagrass shoots (n = 3) were collected, placed in 50 mL beakers containing treatment seawater, and given 10 mins of dark adaptation. Blades received a 5 s saturating light pulse with an excitation irradiance of 3200 µmol photons m^-2^ s^-1^, provided by an array of 6 red (peak wavelength 650 nm) LEDs which focused on a 4 mm diameter point on the seagrass blades. A PIN-photodiode (shielded by a long-pass filter > 720 nm) detected the fluorescence emitted during this saturating pulse, recording the signal every 10 µs for the first 2 ms, then every 1 ms up to 1 s, and then every 100 ms up to 5 s. When plotted on a log_10_ time scale, the FICs followed the O–J–I–P steps of the ‘Kautsky’ curve. The base fluorescence (F_0_) was measured at 0.05 ms (O step), the J step at 1 ms, the I step at 70 ms and the P step (F_M_) was recorded as the maximum fluorescence reached over the 5000 ms sampling period. The rise in fluorescence from O to J represents the reduction of the Q_A_ to Q_A_^–^, while the transition from the J to I step corresponds to further reduction of Q_A_ and, subsequently, Q_B_ [30,31,32]. The final rise to P in the fluorescence transient indicates the filling of the PQ pool [33,34]. FICs were normalised to show the relative variable fluorescence (RVF) [35] at any time t, using the formula RVF = (Ft–F_0_)/(F_M_-F_0_). This calculation allowed for the detection of any changes in the reduced state of Q_A_ [35,36]. The 24, 27 and 30°C treatments did not have any effect on the shape of the FICs, thus the curves from each temperature treatment within each irradiance treatment were pooled for further analysis.

### Photosynthetic and photoprotective pigments

Photosynthetic (chlorophyll *a* and *b*) and photoprotective xanthophyll (violaxanthin, V; antheraxanthin, A; zeaxanthin, Z) and non-photoprotective xanthophyll (β-carotene and Lutein) pigment concentrations of seagrass blades were determined using reverse-phase high-performance liquid chromatography (HPLC), with slight modification from [37]. At the completion of the experiment (n = 3), a seagrass blade was removed from the plant, photographed for surface area determination, stored on ice and then snap frozen in liquid nitrogen until further analysis. Subsequently, blades were placed in 10 mL of HPLC-grade 100% acetone and tissue disrupted using an ultrasonic probe (Sonic and Material Inc. USA; Model-VC50T; 50W, 20 KHz) for 30 s. Samples were kept in the dark and on ice during sonication. The acetone solution was vortexed for 30 s, kept in the dark at -20°C for 12 h, vortexed for a further 30 s, filtered through GF/C filter paper (Whatman) and then spun at 4500 × *g* in a centrifuge for 10 min. 2 ml of the supernatant extract was passed through a 0.2 µm PTFE 13 mm syringe filter (Micro-Analytix Pty Ltd) and placed into an amber HPLC glass vial. The vials were loaded into the auto-sampler and maintained at 4°C and in darkness. Alternate samples of buffer (28 mM tetrabutyl ammonium acetate) and sample/standard were drawn into the sample loop (150 µL buffer, 75 µL sample, 75 µL buffer, 75 µL sample, 150 µL buffer = total of 525 µL) and then injected into an Eclipse XDB C_8_ column (3.4 µm particle size, 150 x 4.6 mm; Agilent Technologies, Australia) and maintained at 55°C with a constant flow rate of 1.1 mL min^-1^.

Pigments were separated using ‘sandwich injection’ where vials of buffer and sample/standard were placed alternately. A linear elution gradient of 95:5% of solvent A (30:70 [w:v] 28 mM tetrabutyl ammonium acetate : methanol [100%]) (pH = 6.5) to solvent B (Methanol [100%]) was applied up to 22 min, with an isocratic hold of 5:95% A:B from 22-29 mins, a return to initial conditions by 31 mins and a further 9 mins of 95:5% A:B, allowing the column to equilibrate before starting the next injection cycle. Analyses were performed using a PC-interfaced HPLC system (Waters, Australia) with a pigment absorbance spectrum measured from 270-700 nm using a photodiode array detector with a 4.3 nm bandwidth. Calibration and quality assurance was performed by using external calibration standards of each pigment (DHI, Hǿrsholm, Denmark). Empower Pro 2 software quantified chlorophyll *a* concentrations at 665 nm, and all other pigments at 450 nm through peak integration. Chlorophyll a, chlorophyll b and β-carotene pigment concentrations were determined relative to seagrass blade surface area (µg cm^-2^). The epoxidation state, EPS (a measure of the proportion of the xanthophyll cycle pool that is present in the de-epoxidised form relative to the epoxidised form) was calculated as [EPS=(Z+0.5A)/(V+A+Z)] [38].

### Seagrass survivorship, biomass, and morphology

In order to measure changes in biomass and morphology characteristics related to temperature and light manipulation, seagrasses were closely monitored over the course of the experiment. The total number of living shoots (*S*) in each tank was recorded prior to the commencement of the experiment and was counted periodically thereafter (days 21, 42, 77 and 91), giving the shoot mortality rate for each treatment. As harvesting of shoots was destructive to the experimental units, whole shoots were only collected at the end of the experiment. Weekly, the number of floating dead leaves in each tank was counted to determine the leaf shed rate for each treatment. At the conclusion of the experiment, dry above-ground (sheaths and leaves) and below-ground (roots and rhizomes) biomass was oven-dried (60°C for 24 h) and weighed for all shoots. To assess leaf morphology, three haphazardly-selected leaves from each sampling unit were photographed and their area calculated using image software (ImageJ; http://rsb.info.nih.gov/ij/).

### Length of study and death of 32°C treatment

Our experiment was conducted over a three month period (approximate length of growing season). Although shoot density declined throughout the experiment, all surviving replicates were still in good photosynthetic condition (ΔF/F_M_’ > 0.6) at the end of the experiment. The exception to this was the 32°C treatments that had 100% mortality after 42 days from time zero. For the purposes of analysis the 32°C were included in an initial analysis to show the effects of the different temperature treatments. As no interaction occurred between light and temperature in the initial analysis, a second analysis was conducted excluding the 32°C treatments for a clearer understanding of the shading effects in the temperature range that seagrasses can survive. Mortality was also experienced in single replicates of 25% shade + 27°C and 0% shade + 30°C; resulting in only two replicates of these treatments and these were excluded from the analysis resulting in an unbalanced experimental design.

### Statistical analyses

To examine the effect of temperature and light on the effective quantum yield (ΔF/F_M_’) of seagrasses we used a two-way Repeated Measures Analysis of Variance (RM ANOVA) design. Initial analysis was conducted with all treatments included and then followed with analysis with the 32°C treatment excluded to determine the main and interactive effect of temperature below the survival threshold. The assumption of sphericity was tested using Mauchleys test and where violated the F value for within group variation was adjusted using the Greenhouse-Geiger correction. Differences in the effects of temperature and light on FICs, leaf width, leaf length, shoot biomass, above:below ground biomass ratio, and pigment concentrations at the end of the experiment were examined using a two-way ANOVA. The assumption of homogenous variances was tested using Levene’s test and graphical examination of residual plots for both RM ANOVA and ANOVA analyses. Data were log-transformed when necessary to improve the assumptions of the ANOVA. Where appropriate, ANOVAs were followed by *a posteriori* Tukeys tests to identify significant differences among means. Data can be accessed via the through the Tropical Data Hub at James Cook University - https://research.jcu.edu.au/researchdata/default/detail/f790117b05fdd500b8e4e5263bb0b01d/

## Results

### Chlorophyll fluorescence

**Figure 2:**
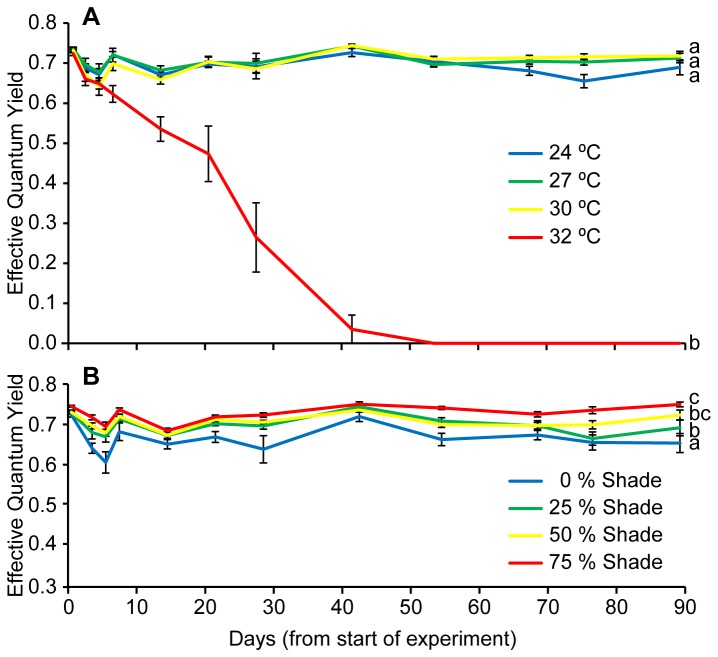
Photosynthetic efficiency. Effective quantum yield of a) different temperature treatments and b) different shade treatments (data pooled from the three surviving temperatures), over the duration of the experiment.

Effective quantum yield (ΔF/F_M_’) of PSII showed a significant interaction between time and temperature (*F*_8.3,_ 83.1= 33.9, *p* < 0.001, Table A in File S1) and significant difference in temperature treatments (*F*_3, 30_= 247.1, *p* < 0.001, Table A in File S1) which was primarily driven by the relatively rapid decline in photosynthetic yield of the 32°C treatment resulting in total mortality after 6 weeks (Figure 2a). When removing this treatment from the analysis, no significant difference was evident among the remaining three temperatures (*F*_2, 22_= 2.46, *p* = 0.109, Table B in File S1), however, there was a significant difference among shade treatments (*F*_3, 22_= 13.5, *p* < 0.001, Table B in File S1) with high shade having the greatest ΔF/F_M_’ (SNK, *p* < 0.05, Figure 2b).

**Figure 3:**
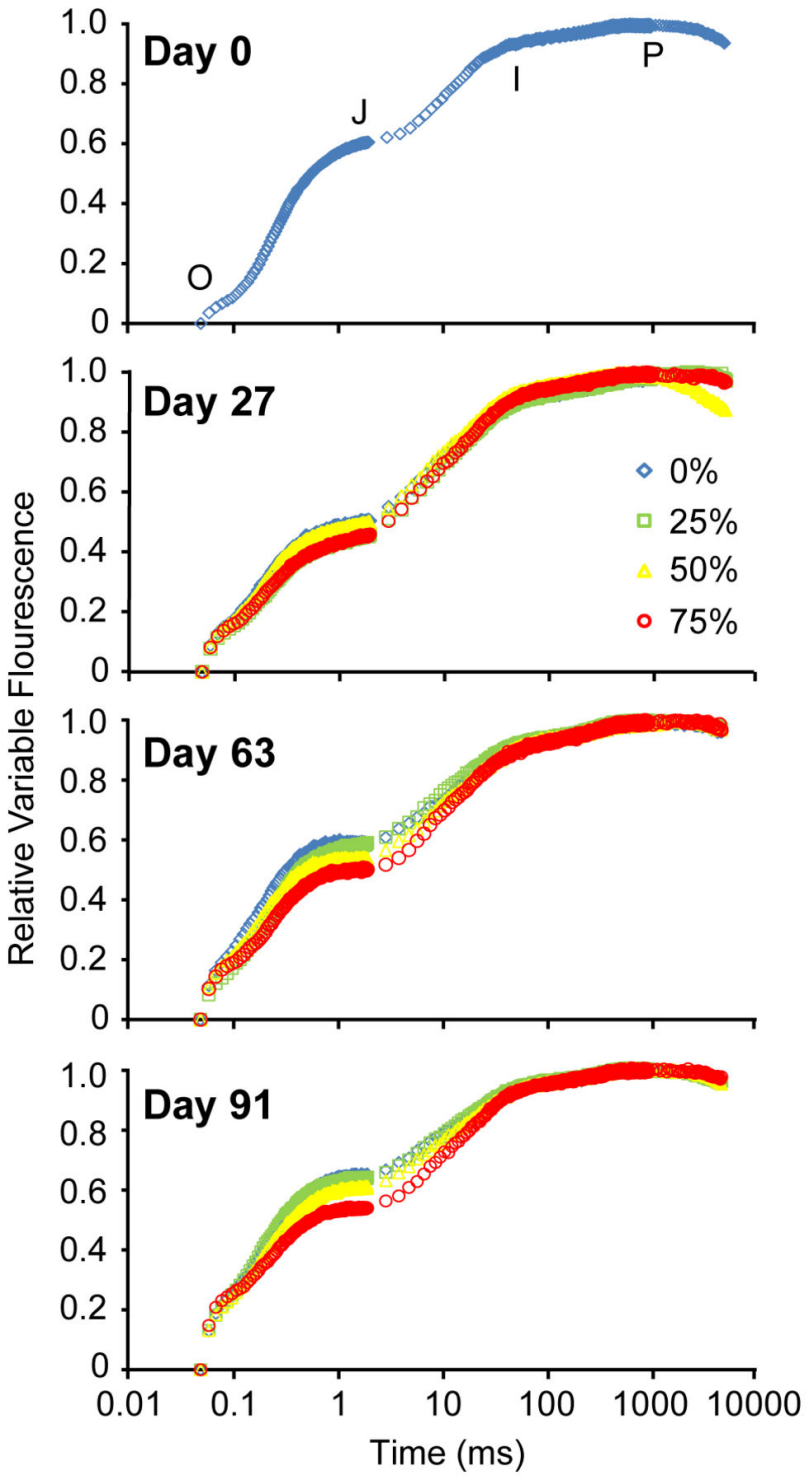
Relative variable fluorescence. The relative variable fluorescence (F_t_-F_0_)/(F_M_-F_0_) of fast induction curves measured on days a) 1, b) 27, c) 53 and d) 92 in the 0, 25, 50 and 75% shade treatments. Each transient represents the average of three temperature treatments (24, 27, 30°C) (*n* = 3). The O, J, I and P steps are marked on the transient from Day 1.

Figure 3 shows FICs in each irradiance treatment (temperature is pooled) at the four measuring time points. Differences in the amplitude of the J step (Table C in File S1) were found on days 53 (*p* = 0.005; Figure 3c) and 92 (*p* = 0.020; Figure 3d), although no differences were found at this point along the FICs on day 27 (*p* = 0.102; Figure 3b). At the two latest time points (days 53 and 92), the amplitude at the J step was significantly lower in the 75% shade treatment compared to both the 0 and 25% shade treatments.

### Photosynthetic and photoprotective pigments

**Figure 4:**
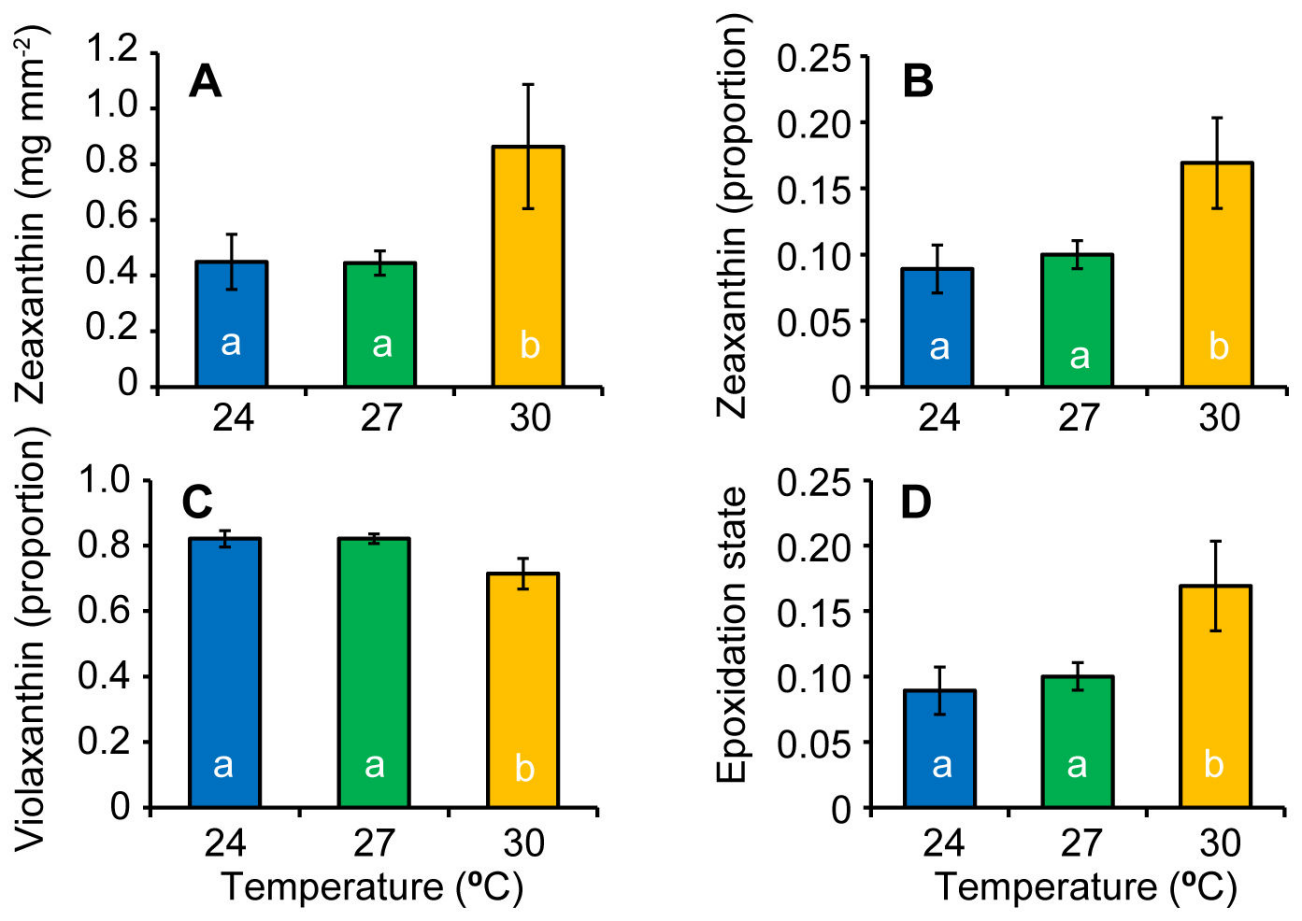
Photoprotective pigments. Photoprotective pigments for surviving temperature treatments for the concentration of a) zeaxanthin, the relative concentrations of b) zeaxanthin and c) violaxanthin and d) the de-epoxidation state.

There were no interactions between temperature and light and no effect of shading for any of the measured photoprotective or photosynthetic pigments (Tables D & E in File S1). Temperature was significantly different among treatments for zeaxanthin concentration (*F*_2, 22_ = 3.52, *p* = 0.047, Table E in File S1) and the relative concentrations of zeaxanthin (*F*_2, 22_ = 3.91, *p* = 0.047, Table F in File S1) and violaxanthin (*F*_2, 22_ = 4.77, *p* = 0.019, Table F in File S1). The epoxidation state was also significantly different among surviving temperature treatments (*F*_2, 22_ = 3.91, *p* = 0.035, Table F in File S1). For both total zeaxanthin concentration and ratio of zeaxanthin relative to the total xanthophyll pool, the 30°C treatment was significantly higher than the two lower surviving temperatures (Figure 4a & b). In contrast, the concentration of violaxanthin relative to the xanthin pool was significantly lower in the 30°C treatment than the two cooler surviving temperature treatments (Figure 4c). Overall, the epoxidation state was significantly higher in the 30°C treatment than the 24 and 27°C treatments (Figure 4d).

### Seagrass survivorship, biomass and morphology

**Figure 5:**
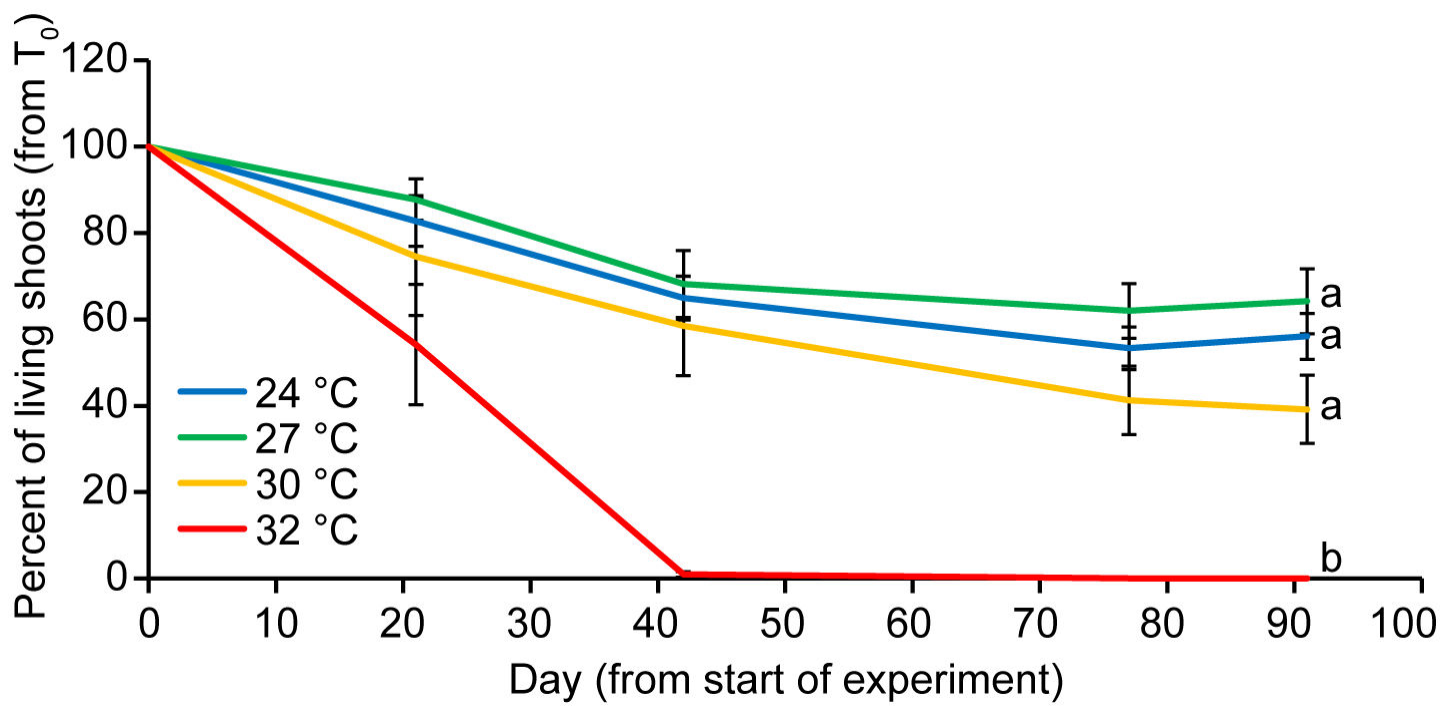
Shoot mortality. The proportion of surviving shoots (mean ± 1 SE) over time for temperature treatments.

The proportion of surviving shoots showed a significant interaction between time and temperature (*F*_6.4,_ 48.9= 7.96, *p* < 0.001, Table G in File S1) and significant difference in temperature treatments (*F*_3, 23_= 14.77, *p* < 0.001), which was primarily driven by the decline in shoot survivorship of the 32°C treatment resulting in total mortality after 6 weeks (Figure 5). The rates of leaf shedding for 24-30°C treatments were identical to those measured in the natural population (0.06 ± 0.011 leaves shoot^-1^ d^-1^, (Gruber – data not shown). However, leaf shed rates for the 32°C treatment were elevated to 0.12 ± 0.041 leaves shoot^-1^ d^-1^ (mean ± SD).

**Figure 6:**
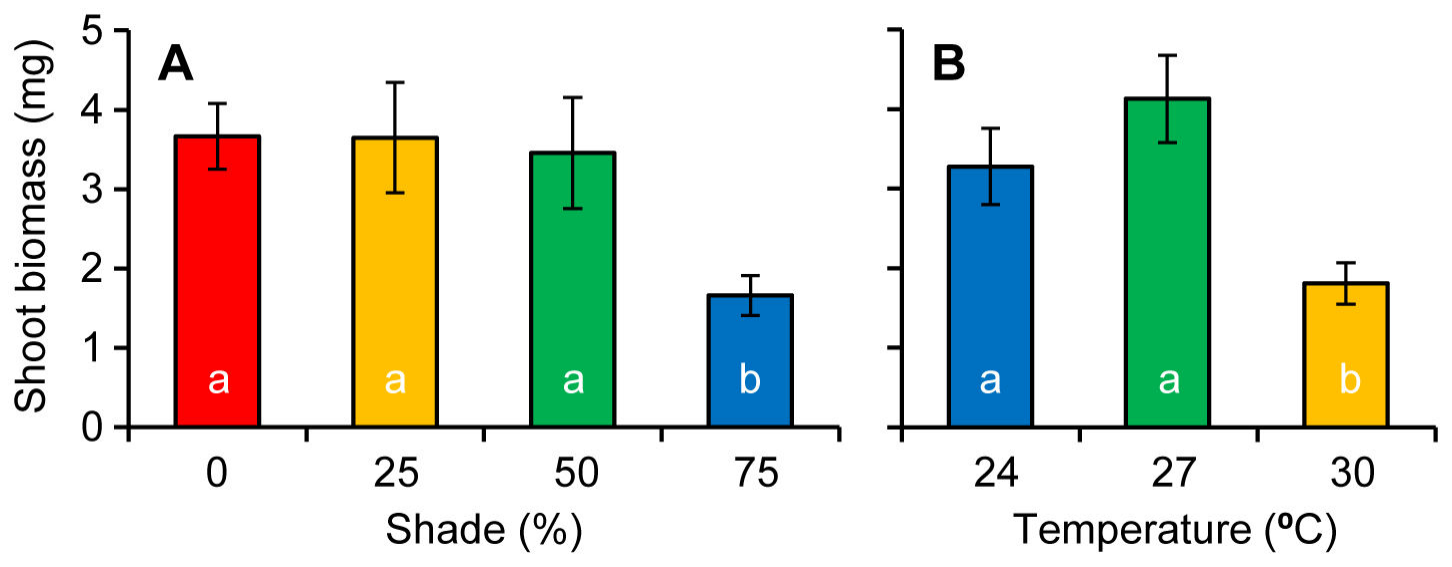
Shoot biomass. Shoot biomass at the end of the experiment among a) different shade treatments and b) the three surviving temperatures treatments.

There was no interaction between temperature and shade with regard to the biomass of individual shoots at the end of the experiment; however, significant main effects were detected for both factors (Shade: *F*_3, 21_= 7.9, *p* = 0.001, Temperature: *F*_2, 21_= 12.9, *p* < 0.001, Table H in File S1). The shading treatment had significantly lower shoot biomass than all other treatments (*SNK p* < 0.5, Figure 6a), while the final biomass of the 30°C treatment had significantly lower biomass than the two lower temperature treatments that survived the duration of the experiment (*SNK p* < 0.5, Figure 6b).

**Figure 7:**
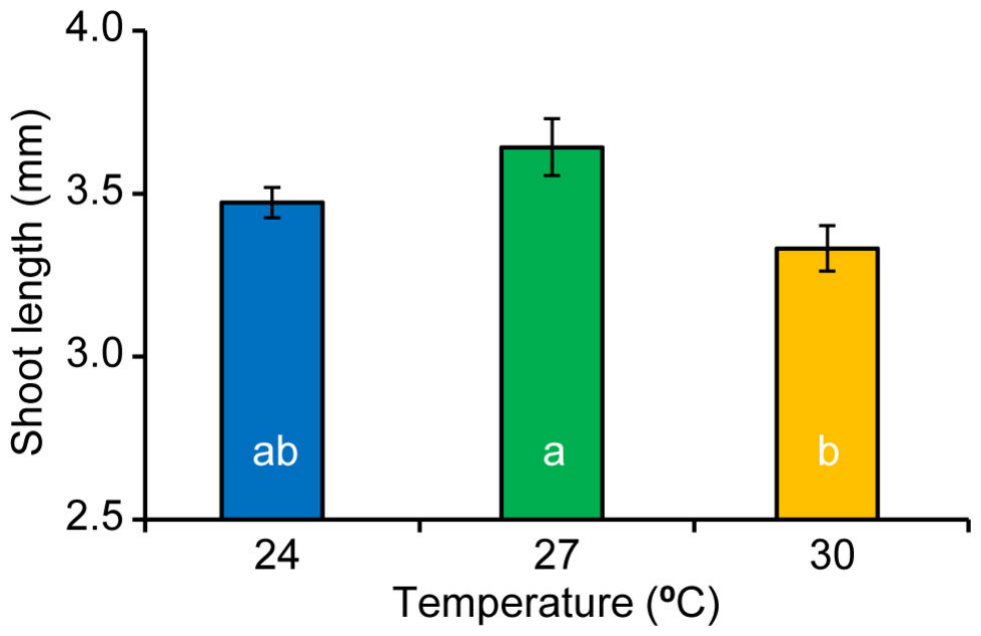
Seagrass canopy height. Seagrass shoot lengths of the three surviving temperature treatments and the culmination of the experiment.

Seagrass leaf lengths were not influenced by the interaction of light and temperature (*F*_6, 22_ = 1.25, *p* = 0.320, Table H in File S1) or shade treatments (*F*_3, 22_ = 2.62, *p* = 0.076); however, there was a significant temperature effect among treatments (*F*_3, 22_ = 6.4, *p* = 0.006). The 27°C treatment had significantly longer shoot lengths than the 30°C treatment, but not the 24°C treatment (*SNK*, *p* < 0.05, Figure 7).

**Figure 8:**
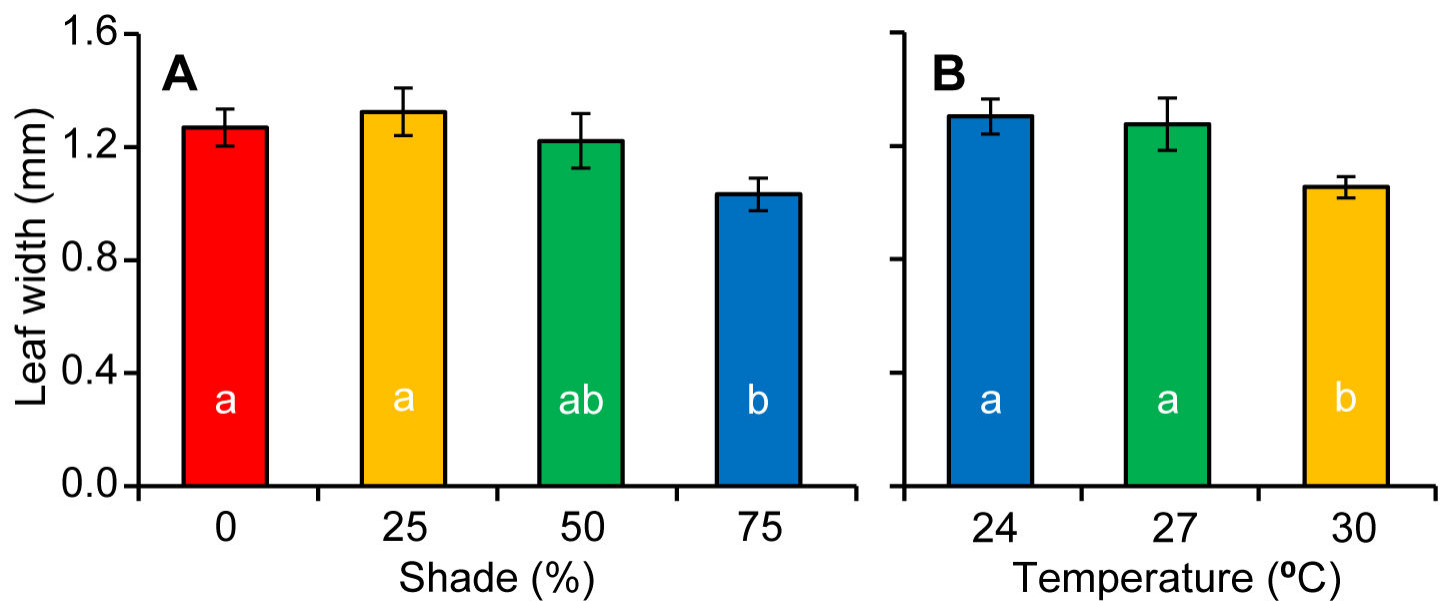
Seagrass leaf widths. Leaf widths (Mean ± 1 S.E.) at the end of the experiment among showing a) different shade treatments and b) the three surviving temperatures treatments.

There was no significant interaction between temperature and shade (*F*_6, 22_ = 0.468, *p* = 0.824, Table H in File S1) on the width of seagrass leaves; however, both main effects showed significant differences among treatments (Temperature: F_3, 22_ = 247.1, *p* = 0.009; Shade: F_2, 22_ = 4.12, *p* = 0.018). Of the surviving temperature treatments, 24 and 27°C had significantly wider leaves than 30°C (SNK, *p* < 0.05, Figure 8a), while high light treatments (0 and 25 % shade) had significantly wider leaves than the lowest light treatment (SNK, *p* < 0.05, Figure 8b).

**Figure 9:**
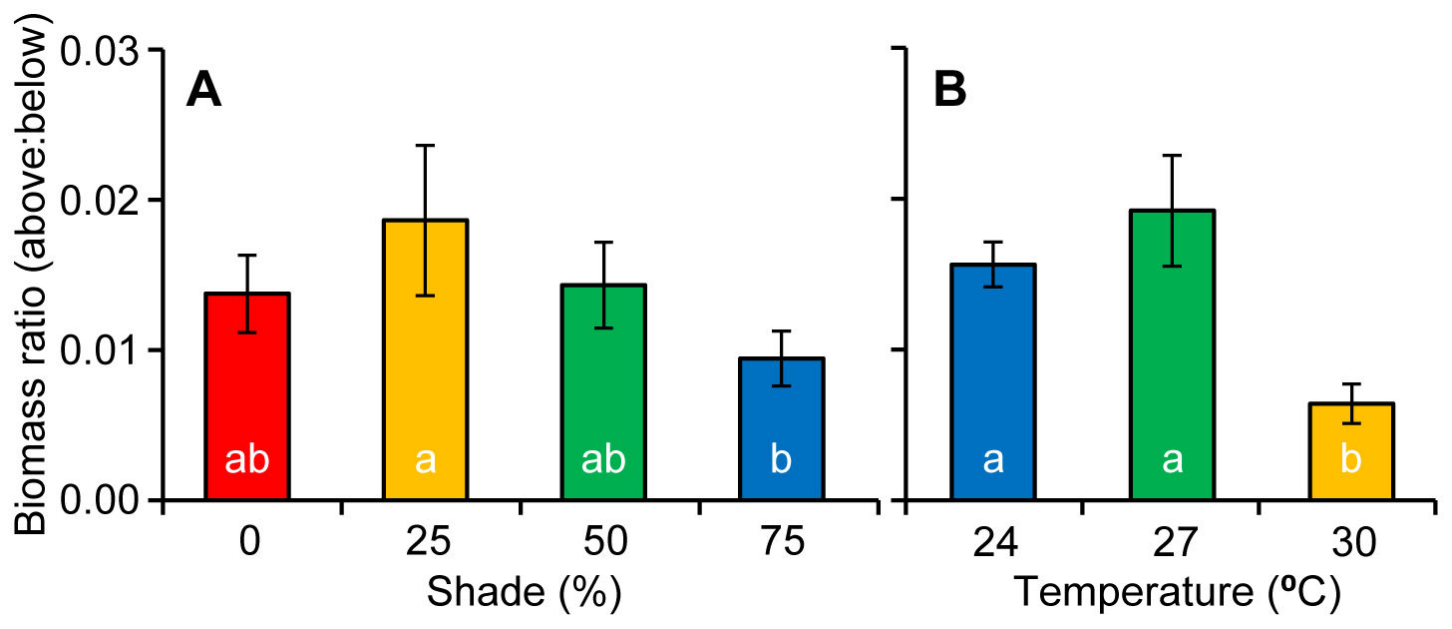
Resource partitioning of seagrass tissue. The ratio of above to below ground seagrass biomass (Mean ± 1 S.E.) at the end of the experiment among a) different shade treatments and b) the three surviving temperatures treatments.

The ratio of above/below ground biomass at the end of the experiment showed no interaction between temperature and shade (*F*_6, 22_ = 2.33, *p* = 0.680); however, temperature (*F*_3, 22_ = 13.2, *p* < 0.001) and shade (F_2, 22_ = 4.12, *p* = 0.017) both caused significant differences in the ratio of above/below ground biomass among treatments (Table H in File S1). Of the surviving temperature treatments, the 27°C treatment had a significantly higher ratio than the 30°C treatment, but not the 24°C treatment (*SNK*, *p* < 0.05, Figure 9a). The 75% shade treatment had a significantly lower ratio than all treatments except the 50% treatment (*SNK*, *p* < 0.05, Figure 9b).

## Discussion

### Temperature effects

Temperature had a strong and immediate effect on short-term survival of *Zostera muelleri*. Seagrasses experiencing 32°C showed complete mortality, displaying a steady decline in effective quantum yield of PSII (ΔF/F_M_’), falling below measurable levels by day 42. Rates of leaf shedding at 32°C were also double that in other temperature treatments and the field-validated rate. Photosynthetic efficiency remained steady throughout the experiment among the 24, 27 and 30°C treatments. There was also a stabilisation of shoot numbers among these remaining treatments by the conclusion of the experiment. Despite having similar EQY, these treatments showed differences in biomass partitioning and morphology after 3 months. The 30°C treatment displayed significantly reduced above-ground biomass and smaller leaves compared to the 24, and 27 treatments.

The temperature threshold of ~30°C for *Zostera muelleri* is similar to a well-studied temperate species in the northern hemisphere, *Z. marina* [39,40,41]. Although studies of *Zostera muelleri* typically occur in warmer climates [18,42], thresholds from these regions are also similar to the one described in the present study. Our results are comparable to the findings of [42] who studied *Z. muelleri* in a tropical environment (^≈^ 1500 km N of our study site) and found a similar trend using leaf growth rates and above ground biomass; although unlike our results, their study showed no significant differences between 27 and 30°C treatments. This suggests *Z. muelleri* may have the ability to adapt its photosynthetic efficiency within the local temperature range but little capacity to survive once the temperature threshold for this species is exceeded. Higher temperatures cause an increase in plant respiration, which creates a carbon imbalance where the use of carbon for respiration begins to exceed the amount fixed through photosynthesis [20,43]. In our study, high rates of leaf loss may have been an attempt by plants to restore their carbon balance by reducing above-ground tissues, which have higher respiratory demands than below ground tissues [44]. Reduced biomass accumulation in warmer (30°C) treatments after 90 days suggests that respiration was slightly elevated at these temperatures and could cause mortality over a longer time frame. Another explanation could be that greater hydrogen sulphide production at higher temperatures creating toxic levels in sediment pore water [45,46].

Although the concentration of photosynthetic pigments was not different between treatments at the end of the experiment, the up-regulation of photoprotective pigments was observed at 30°C, with a greater epoxidation ratio due to zeaxanthin production. The change in photoprotective pigments as a result of increasing temperature indicates excess energy was dissipated through xanthophyll cycling, a form of non-photochemical quenching [47,48].

Under moderate heat, violaxanthin content has been linked to increased thermal stability of the photosynthetic apparatus in the form of more fluid thylakoid membrane [49]. Our data also showed under moderate heat and sub-saturating light violaxanthin was photoconverted to zeaxanthin. This makes ecological sense as thermal stress and heat stress are usually combined under field conditions. The fact that the epoxidation ratio did not respond to light levels indicates that the light treatments were not high enough to affect the epoxidation process.

### Light effects

The greatest effect of light was on photosystem II (PSII) photochemical efficiency. Irradiance strongly influenced PSII photochemical efficiency by day 92, with an irradiance-dependent separation of ΔF/F_M_’. Greater levels of shade were effective in maintaining high photochemical efficiencies, while higher irradiance suppressed ΔF/F_M_’. This response is consistent with enhanced light harvesting efficiencies in other seagrass under low light conditions [50]. Similarly, the fast induction curve (FIC) chlorophyll fluorescence measurements revealed that on days 53 and 92, the amplitude at the J step was significantly lower in the 75% shade treatment compared to both the 0 and 25% shade treatments. The lower J step amplitude indicates a greater oxidation of the primary electron acceptor of PSII (Q_A_) [31,32,51], which is likely to have contributed to the higher ΔF/F_M_’ under more shaded conditions. The higher J step in the 0% shade treatment indicates Q_A_ reduction and closure of PSII reaction centres [35,36]. Therefore, at temperatures below thermal thresholds of survival in *Z. muelleri*, light is the driver of photosynthetic condition, with more shaded environments allowing for the persistence of high PSII photochemical efficiencies. Reduced light levels through shading also led to changes in a suite of morphological characteristics. Many of these changes have been previously observed in other seagrass studies (e.g. a reduction in leaf and shoot density [52,53]; and a reduction in the above ground biomass of leaves and stems relative to roots and rhizomes [53]) while a reduction in shoot width was contrary to the findings of other studies that reported no change in width under reduced light conditions [54,55,56]. Reduction of leaf and shoot biomass under low light conditions is a common response in plants and can have a negative impact on carbon fixation, but may also be a photo-adaptive response to reduce self-shading within the canopy [50,57]. Leaf senescence is a common response in higher plants to strong shading when photosynthetic acclimation can no longer maintain a positive carbon balance [58].

Light did not influence xanthophyll pigment concentration or the de-epoxidation state, suggesting that the light treatments applied did not exceed saturating irradiances. Seagrasses were harvested from a site with relatively high water clarity (*k*_*d*_= -0.45 m^-1^), and therefore would have experienced midday irradiances exceeding 1000 µmol photons m^-2^ s^-1^, whereas light levels experienced in our experiment are more representative of turbid or deep-water habitats.

### Future implications for survival and management

Recent studies of multiple stressors in marine environment suggest that stressors generally interact synergistically, indicating that cumulative effects are greater than the addition of individual stressor [27,59]. As seagrasses growing in low light conditions have previously been found to have lower optimum temperatures for photosynthesis [60], plants at high temperatures require more light to maintain a positive carbon balance [50]. However, contrary to expectations, there was scant evidence of a synergistic effect of temperature and light level. An interaction between temperature and light may exist over a wider range of irradiances than our study provided; see 60, however, our results match a lack of interaction was also found in a similar study recently completed in a tropical system [42]. The absence of non-linearity in the temperature and light responses should simplify predictions of seagrass loss and make prevention measures to combat this loss more straightforward than if interactions occurred among stressors.

The thermal threshold for seagrass survival collected from Lake Macquarie, is between 30 and 32°C, evidenced by the rapid mortality of the high temperature treatment in our experiment. During the austral summer months at the site of collection, maximum temperatures reach (and exceed) this thermal threshold on a regular basis (see Figure 1), indicating that Lake Macquarie *Z. muelleri* populations are currently living close to their upper thermal limit. However, the duration of exposure to thermal stress is an important factor to consider in determining the threat of thermal events to seagrasses; currently, seagrasses in the field are only experiencing temperature extremes for several days at a time (i.e. acute stress, typical of a ‘heat wave’), whereas our study is based on chronic exposure, which is more relevant to long-term climate change. Interestingly, chronic temperature increases have occurred in two regions of Lake Macquarie, which receive hot water discharge from coal-fired power stations (Vales Point and Eraring Power Stations). Areas around the largest station (Vales Point) previously occupied by *Z. muelleri* were replaced by *Halophila ovalis* (a smaller, more tolerant seagrass) in 1980 [61] and have failed to re-establish in the last 33 years. The average temperature increase in this region is ~2°C [62]. Both acute and chronic temperature increases are predicted for south-eastern Australia as a result of climate change [25], with average sea surface temperatures in central New South Wales predicted to increase by 0.6 °C by 2030 and by 1.0-2.5 °C by 2070 based on low (B1) and high (A1FI) future emissions scenarios respectively [63]. These increases are likely to cause range shifts (pole ward) and contractions in seagrass extent [40,64,65].

Below this thermal threshold for seagrass survival, our study indicates that changing light conditions are likely to have a greater influence than temperature on seagrass health. Over decades, urbanisation and land clearing in coastal catchments of south-eastern Australia has led to decreases in water quality and a reduction in light levels in many estuaries [66]. Although low light levels seemed to cause increased photosynthetic efficiency, the absence of common photo-adaptive responses in our experiment, such as increases in photosynthetic pigments or increased leaf surface area [50], suggest that *Z. muelleri* may be limited in its ability to adapt to low light environments. It was unexpected that seagrasses survived the duration of this experiment (90 days) under very low light levels (75% shade), though the number of living shoots and shoot biomass significantly declined. This result suggests that chronic decreases in water clarity may take months to years before a measurable effect occurs in the seagrass population. It is important to note that measures of photosynthetic performance were completed on healthy sections of seagrass leaves/shoots, and are representative of this tissue only. As a consequence, the chlorophyll fluorescence results presented here are only an indicator of viable sections of seagrass shoots and do not reflect the condition of the plant as a whole. In order to map the spatial heterogeneity of photosynthetic performance across a seagrass blade, imaging technology is recommended [67].

The stresses faced by seagrass communities in the next several decades will be considerable. There is some indication that genetic diversity may facilitate seagrass populations’ adaptation to change [68], and our results showed seagrass acclimation to high temperature and low light through biomass partitioning. However, the similarity between our results and those of tropical populations of *Z. muelleri* [42] plus evidence from *in situ* chronic temperature increases in Lake Macquarie suggests a lack of ability for this species to adapt above measured thresholds.

In summary, these results demonstrate that populations of *Z. muelleri* in south-eastern Australia (particularly in shallow coastal lakes and lagoons where mixing and flushing is limited) are sensitive to small chronic temperature increases and light decreases. While temperature and light do not interact in a synergistic way, the presence of the two in conjunction will have additive effects that place increased stress on seagrass populations. From a management perspective, the amelioration of the effects of climate change requires a long-term global effort, yet the improvement of water quality is achievable in the short-term at the catchment scale. Stronger controls on nutrient and sediment inputs to coastal systems will immediately improve seagrass condition and likely increase resilience to climate change.

## Supporting Information

Figure S1 **Experimental layout.** The above diagram shows a plan view of the placement of temperature and light treatments in the experiment and the diagram below shows the set up of each individual experimental unit.
(TIF)

File S1 **Analysis of variance results.** Detailed analysis of variance (ANOVA) result tables for physiological and morphological responses of temperature and light treatments during the experiment.
(PDF)
