# Supplementary figures and images for: Physiological and Morphological Responses of the Temperate Seagrass Zostera muelleri to Multiple Stressors: Investigating the Interactive Effects of Light and Temperature
Source: PLoS One. 2013 Oct 4;8(10):e76377. doi: 10.1371/journal.pone.0076377 (PMC3790674; doi:10.1371/journal.pone.0076377)

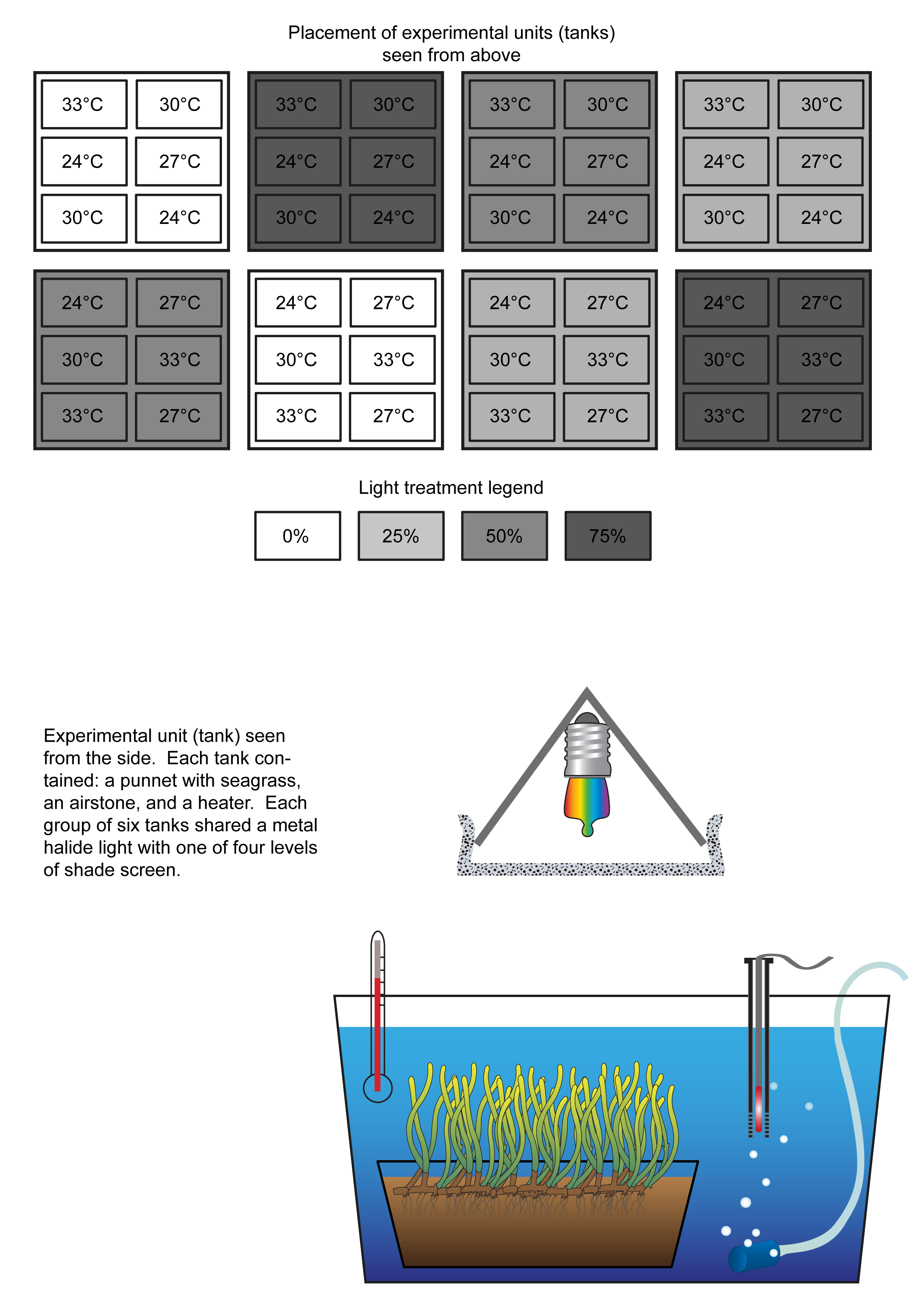

Supplement: Figure S1 — Experimental layout. The above diagram shows a plan view of the placement of temperature and light treatments in the experiment and the diagram below shows the set up of each individual experimental unit. (TIF) [file pone.0076377.s001.tif]
