# Supplementary material for: Physiological and Morphological Responses of the Temperate Seagrass Zostera muelleri to Multiple Stressors: Investigating the Interactive Effects of Light and Temperature
Source: PLoS One. 2013 Oct 4;8(10):e76377. doi: 10.1371/journal.pone.0076377 (PMC3790674; doi:10.1371/journal.pone.0076377)
Supplement: File S1 — Analysis of variance results. Detailed analysis of variance (ANOVA) result tables for physiological and morphological responses of temperature and light treatments during the experiment. (PDF) [file pone.0076377.s002.pdf]

Table A. Repeated measures analysis of variance results for differences among treatments of effective quantum yield of PSII including data from all treatments.

**Source**	**df**	**MS**	**F**	**Sig.**
		Within subject effects		
Day	7.3*	66645	17.94	**<0.001**
Day x shade	22.0*	4533	1.22	0.237
Day x temp	14.7*	8446	2.27	**0.007**
day x shade x temp	44.0*	4418	1.19	0.219
Error (day)	161.4*	3715		
		Between subject effects		
Shade	3	143068	13.50	**<0.001**
Temp	2	26079	2.46	0.109
Shade x temp	6	5392	0.51	0.795
Error	22	10597		

* Degrees of freedom adjusted using Greenhouse-Geiger correction

Table B . Repeated measures analysis of variance results for differences among treatments of effective quantum yield of PSII with data from 33˚ C treatments excluded.

**Source**	**df**	**MS**	**F**	**Sig.**
		Within subject effects		
Day	2.5*	1113116	55.91	**<0.001**
Day x shade	7.4*	16213	0.81	0.584
Day x temp	33.0*	291552	65.07	**<0.001**
day x shade x temp	22.3*	11403	0.57	0.930
Error (day)	74.3*	19908		
		Between subject effects		
shade	3	87031	5.81	**0.003**
temp	3	4780992	319.15	**<0.001**
shade x temp	9	8219	0.55	0.827
Error	30	14980		

* Degrees of freedom adjusted using Greenhouse-Geiger correction

Table C. Analysis of variance results for the J step in the fast induction curves (FICs) derived from PAM fluorometry of PSII in seagrass leaves.

Variable	Factor	df	MS	F	Sig.
**FIC (J-Step) Day 27**	Shade	3	0.02	2.245	0.102
	Error	32	0.01		
**FIC (J-Step) Day 53**	Shade	3	0.02	5.106	**0.005**
	Error	32	0.00		
**FIC (J-Step) Day 92**	Shade	3	0.02	3.754	**0.020**
	Error	32	0.01		

Table D. Analysis of variance results for differences among treatments of photosynthetic pigments in seagrass leaves at the culmination of the experiment.

Source	Factor	df	MS	F	Sig.
**Chlorophyll *a***	Shade	3	48.75	0.132	0.94
	Temperature	2	179.98	0.488	0.621
	Shade x Temp	6	53.42	0.145	0.988
	Error	22	369.09		
**Chlorophyll *b***	Shade	3	38.18	0.577	0.636
	Temperature	2	80.95	1.224	0.313
	Shade x Temp	6	14.2	0.215	0.968
	Error	22	66.12		

Table E. Analysis of variance results for differences among treatments of photoprotective pigments in seagrass leaves at the culmination of the experiment.

Source	Factor	df	MS	F	Sig.
**Neoxanthin**	Shade	3	0.01	0.02	0.997
	Temperature	2	0.08	0.11	0.900
	Shade x Temp	6	0.37	0.52	0.788
	Error	22	0.71		
**Violaxanthin**	Shade	3	0.59	0.30	0.826
	Temperature	2	2.08	1.06	0.364
	Shade x Temp	6	0.31	0.16	0.986
	Error	22	1.97		
**Antheraxanthin**	Shade	3	0.01	0.10	0.957
	Temperature	2	0.15	1.83	0.184
	Shade x Temp	6	0.06	0.80	0.581
	Error	22	0.08		
**Zeaxanthin**	Shade	3	0.15	0.44	0.730
	Temperature	2	1.21	3.50	**0.047**
	Shade x Temp	6	0.24	0.71	0.648
	Error	22	0.35		
**Lutein**	Shade	3	0.08	0.02	0.996
	Temp	2	2.21	0.55	0.586
	Shade x Temp	6	0.8	0.20	0.974
	Error	22	4.04		
**β Carotene**	Shade	3	0.39	0.19	0.901
	Temperature	2	0.29	0.14	0.870
	Shade x Temp	6	0.25	0.12	0.992
	Error	22	2.04		

Table F. Analysis of variance results for differences among treatments in xanthophyll pool in seagrass leaves at the culmination of the experiment.

Source	Factor	df	MS	F	Sig.
**Total xanthophyll (V+A+Z)**	Shade	3	0	0.207	0.89
	Temp	2	0	2.983	0.071
	Shade x Temp	6	0	0.943	0.485
	Error	22	0		
**Epoxidation state**	Shade	3	0.17	0.522	0.671
	Temp	2	1.26	3.906	**0.035**
	Shade x Temp	6	0.25	0.758	0.61
	Error	22	0.32		
**Zeaxanthin Relative concentration Z/(V+A+Z)**	Shade	3	0.17	0.522	0.671
	Temp	2	1.26	3.906	**0.035**
	Shade x Temp	6	0.25	0.758	0.61
	Error	22	0.32		
**Antheraxanthin Relative concentration A/(V+A+Z)**	Shade	3	0	0.207	0.89
	Temp	2	0	2.983	0.071
	Shade x Temp	6	0	0.943	0.485
	Error	22	0		
**Violaxanthin Relative concentration V/(V+A+Z)**	Shade	3	0.02	0.774	0.521
	Temp	2	0.13	4.769	**0.019**
	Shade x Temp	6	0.04	1.321	0.289
	Error	22	0.03		

Table G. Repeated measures analysis of variance results for rate of leaf loss.

Source	df	MS	F	Sig.
Tests of Within-Subjects Effects				
Day	2.1*	47569	117.53	**<0.001**
Day x shade	6.4*	469	1.16	0.343
Day x temp	6.4*	3221	7.96	**<0.001**
day x shade x temp	19.2*	407	1.01	0.470
Error (day)	49.0*	404		
Tests of Between-Subjects Effects				
Shade	3	1522	1.12	0.361
Temp	3	20092	14.78	**<0.001**
Shade x temp	9	911	0.67	0.727
Error	23	1359		

* Degrees of freedom adjusted using Greenhouse-Geiger correction

Table H. Analysis of variance results for differences among treatments in morphological traits of seagrass at the culmination of the experiment.

Source	Factor	df	MS	F	Sig.
**Shoot biomass**	Shade	3	1.382	7.902	**0.001**
	Temp	2	2.253	12.879	**<0.001**
	Shade x Temp	6	.077	.443	0.842
	Error	21	.175		
**Leaf length**	Shade	3	.118	2.622	0.076
	Temp	2	.288	6.426	**0.006**
	Shade x Temp	6	.056	1.250	0.320
	Error	22	.045		
**Leaf width**	Shade	3	.111	4.124	**0.018**
	Temp	2	.157	5.811	**0.009**
	Shade x Temp	6	.013	0.468	0.824
	Error	22	.027		
**Above / below ground ratio**	Shade	3	.000	4.212	**0.017**
	Temp	2	.001	13.198	**<0.001**
	Shade x Temp	6	.000	2.327	0.068
	Error	22	.000		
